# What The Cognitive Neurosciences Mean To Me

**DOI:** 10.4103/0973-1229.32160

**Published:** 2007

**Authors:** Alfredo Pereira

**Affiliations:** *São Paulo State University (UNESP) - Institute of Biosciences - 18618-000 - Botucatu - SP, Brazil Email: apj@ibb.unesp.br*

**Keywords:** *“Covering law” model*, *cognitive molecular neurobiology*, *cognitive neuroscience*, *embedded and embodied cognition*, *homeoresis*, *philosophy of mind*, *reductionism*, *structuralism*, *structuralist-naturalistic approach*

## Abstract

Cognitive Neuroscience is an interdisciplinary area of research that combines measurement of brain activity (mostly by means of neuroimaging) with a simultaneous performance of cognitive tasks by human subjects. These investigations have been successful in the task of connecting the sciences of the brain (Neurosciences) and the sciences of the mind (Cognitive Sciences). Advances on this kind of research provide a map of localization of cognitive functions in the human brain. Do these results help us to understand how mind relates to the brain? In my view, the results obtained by the Cognitive Neurosciences lead to new investigations in the domain of Molecular Neurobiology, aimed at discovering biophysical mechanisms that generate the activity measured by neuroimaging instruments. In this context, I argue that the understanding of how ionic/molecular processes support cognition and consciousness cannot be made by means of the standard reductionist explanations. Knowledge of ionic/molecular mechanisms can contribute to our understanding of the human mind as long as we assume an alternative form of explanation, based on psycho-physical similarities, together with an ontological view of mentality and spirituality as embedded in physical nature (and not outside nature, as frequently assumed in western culture).

## Introduction

The modern western scientific view of the world is based on a separation of body and mind. Recently, this separation has been questioned by a growing number of scientists, leading to the emergence of new, inter and trans-disciplinary areas or research.

Neuroscience is one such multi-disciplinary area, composed of several approaches that study brain structure and function, such as neuroanatomy, neurophysiology, neuropharmacology and systems neuroscience. The Cognitive Neurosciences (CN) comprise of an interdisciplinary area of research that attempt to elucidate the relations of brain structure/function and cognitive processing: therefore providing a link between the study of the brain and the study of the mind. Successful research in this area, mainly from neuroimaging studies, is expected to address philosophical problems concerning perception, thinking, language, intentionality and the construction of the concept of Self, areas which also are of direct interest to Psychiatry.

## A personal trajectory

My first contact with the Cognitive Neurosciences was by means of Gazzaniga's (1995) classical collection, when I realized that old philosophical questions concerning the human mind could be approached by means of scientific methodology. Having a academic formation in the Philosophy of Science, the Cognitive Neurosciences were interesting to me for two reasons: first, I could make a philosophical analysis of the concepts and methodologies used in the area; and second, I could critically evaluate if the results could afford a better understanding of the human mind.

In 1996, the Brazilian funding agency FAPESP sponsored me for a two-year postdoctoral Fellowship period at the Massachusetts Institute of Technology (MIT). This was in the Department of Brain and Cognitive Sciences, under the supervision of Dr. Stephan L. Chorover. It was a great opportunity to learn more deeply about this new interdisciplinary area and develop theoretical research about its foundations. One of my first realisations was that the area was not created by Gazzaniga's effort, but had a long history since the 1960s, when the Neurosciences Research Programme was created at MIT.

In 1996 and 1997, I attended the Cognitive Neuroscience Society meetings in Boston and San Francisco (USA). At this time, researchers were still asking themselves about the nature of research in this area. Some of them were inclined to define CN as part of the neurosciences that investigate cognitive processes, while others defined it as the part of cognitive science that elaborates on biologically-inspired explanatory models. This division was also present at the MIT's Department, with two different groups working at different places and with different perspectives.

To be frank, I was not satisfied with these two main currents, because they were used to making simplistic assumptions about the brain activities that support consciousness, the so-called “neural correlates of consciousness” (I do not like to use this expression since I have good reasons to believe that glial cells also participate in consciousness). Neuroscientists doing brain imaging research were only looking for which areas became more activated for each kind of cognitive task. Cognitive scientists, on the other hand, elaborated computational models with artificial neurons performing arithmetical operations, focusing on their connectivity patterns. Different patterns of connectivity were taken by both currents as explanations of different mental phenomena, but this kind of explanation did not throw new lights on mental life (see Thagard, 2003).

My personal trajectory bent towards a third direction: theoretical research about molecular neurobiological mechanisms that supports consciousness, with a focus on psychiatric phenomena as the best available pathway to understand how the brain generates mental activity and consciousness.

I must explain what dissatisfied me about the other two approaches. They have been successful according to scientific standards and eventually they were integrated into a well-accepted paradigm in the area. However, they only scratch the surface of the philosophical problem of relating brain and mind. Cognitive molecular neurobiology, in turn, although also being a scientific area obeying rigid experimental procedures, has brought a richness of details about the workings of the brain, allowing the construction of new hypotheses about the biophysical mechanisms that support mental life.

## Cognitive Neuroscience and Molecular Neurobiology

Recent advances in neurobiology have provided explanations of mental processes - as memory and perceptual recognition - based on genetic and molecular structures. The 2004 Medicine Nobel Prize confirms a previous tendency to value the discovery of molecular mechanisms that support mental activities. (For information about Nobel Laureates, see http://nobelprize.org/ for information on 2004 Laureates in Physiology or Medicine, see )http://nobelprize.org/nobel_prizes/medicine/laureates/2004/index.html. The work of E. Kandel, P. Greengard and E. Carlsson, awarded in 2001, focused on microstructures responsible for learning, memory and the effect of psychoactive substances. The work of the 2004 laureates, R. Axel and L.B. Buck and the line of research they have contributed to construct, reveal a connection between the activity of specific molecules (genes, protein receptors) and mental experiences as odour recognition (see Leon and Johnson, 2003) and pain (see Reilly *et al.*, 2004).

Such a choice of Nobel Laureates indicates how the discoveries - about a close relation between our molecular structure and our mental functions - are important (and surprising) for the scientific community. This relation was already evident in Medicine, where the search for the Human Genome was regarded as a crucial step in the understanding and treatment of several kinds of illnesses. However, the realms of the human mind were usually assumed to be beyond the biochemical organization of the brain, possibly because of religious beliefs holding that our mental life is the property of a spiritual soul, independent of the body and able to survive after biological death.

Today, besides relating mental functions with differentially activated brain areas or putative neural networks, molecular neurobiologists and biophysicists are beginning to relate them with ions and molecules composing signaling pathways. When the properties of the elements of a system count as a part of the explanation of emergent phenomena in this system, a *structuralist* approach is adopted: *the structure of our brains determine the possibilities of our minds*. If we had different genes and then different olfactory receptors, the odours we perceive would be different. Of course we still don't know the complete pathway that leads from molecular to mental properties, but the time is near when this question can be raised and answered.

It is important to note that the structure of living beings is not *static*. In the study of a system that evolves in time - a dynamic system - it is necessary to consider a structure with components that vary along time, as in the classical concept of *homeoresis* advanced by Waddington (1977). Homeoretic systems undergo an evolutionary process that changes not only their functions, but also their structure. For instance, the development of an embryo is a process where both structure and function change, leading to the emergence of new properties. Therefore, the idea of *structuralism* does not imply the existence of a static structure generating a finite set of functions. It is compatible with an evolving structure generating a potentially infinite set of functions.

The emergence of mental activity from brain processes - interacting with the whole body and the environment - is a typical case of *strong emergence* (Stephan, 1999), when the resulting states of a physical process cannot be completely deduced, even ‘a posteriori’, from the laws/principles governing the system. The structuralist approach contrasts with current projects in the areas of Artificial Intelligence (AI) and Artificial Life (AL), which ignore structural constraints governing the emergence of mental activity. This outlook causes a limitation in their approach to life and mentality, since the knowledge of the structure of a system can support inductive predictions about its behaviour. Although the efforts of AI and AL may be valid according to their technological purposes, the explanations of life and mentality that this kind of research can offer are severely limited by the explanatory resources that they use.

## Different Views of Scientific Explanation

Molecular Neurobiology models brain activity in terms of interacting ions and molecules. The biophysical properties of ions and molecules are assumed to generate, by means of self-organizing processes (including the interaction with the body and environment), emergent mental properties as learning, memory, attention, affection, emotion and consciousness. Inspired by the empirical results achieved by this approach, I understand that a new conception of scientific explanation is needed to account for emergent properties.

Such an understanding is not based on a *reductionist* approach (as the one defended by Bickle, 2003), of *deducing* mental phenomena from neuroscientific theories and bridge principles. Structuralism is an alternative view that considers the properties of the structure in order to evaluate the *similarity* of structural functions with mental functions. The reasoning based on similarities is able to support inductive inferences from biophysical to mental structures that are not possible in a syntactic-deductive modality of reasoning. There is a close rapport between judgment of similarity and inductive inferences, the first being more fundamental in human thinking and used to solve the classical problems of induction (a detailed argument on this purpose is presented by Gärdenfors, 2000).

I give four examples to illustrate how the reasoning based on psycho-physical structural and functional similarities operate in the context of explaining cognitive phenomena from molecular neurobiology:

1. First, animals with a deficit in the protein CaMKII (Calmodulin-dependent Protein Kinase II) activity display a deficit in memory formation. This reasoning is *induction by vicariance*: the lack of a function *f* in the brain implies the lack of a function *f′* in the mind.
2. A second example is that a decrease in serotonin levels predicts the onset of depression. This reasoning is *induction by similarity*: once serotonin is a neuromodulator that increases the efficacy of synapses, a decrease in serotonin levels would also decrease the efficacy of synaptic communication. Although we don't know exactly what synaptic communication has to do with *mood*, a mental phenomenon, we can find a similarity between serotonin decrease and a decrease in mental disposition (I do not believe low serotonin to be the *cause* of depression; for my view on this subject, see Pereira *et al.*, 2006).
3. The third example is also a case of inductive reasoning, based on an experiment that produced an *increase* in molecular function. It is well known that the membrane receptor NMDA is involved in the capacity of associative learning. Genetically modified mice with over-expression of the NMDA receptor are predicted to display improved learning capabilities. This reasoning is based on the function of the NMDA receptor in the single cortical neuron, where it works as a coincidence detector, providing neuronal excitation upon receiving two excitatory pulses in a narrow time window. Associative learning is a mental function that consists basically of connecting different stimuli. It is not the case that the pulses received by the NMDA receptor at each neuron correspond to the stimuli to be associated; however, its physiological function has some degree of similarity with the mental function (for a more detailed analysis of glutamatergic mechanisms involved in perceptual learning, see Pereira Jr, 2006).
4. The fourth example is a case when *increasing* the quantity of one kind of molecular component leads to a *decrease* of mental activity. Also in this case there is *induction by similarity*, but this time with inverse proportionality. It is well known that the transmitter GABA and its receptors have a physiological function of inhibiting neuronal activity. The intake of substances that perform the same function of GABA is used in Psychiatry as tranquillizer, in the treatment of anxiety and psychoses. Anxiety is a mental phenomenon, having properties that cannot be deduced from biophysical processes in the brain. However, we can find a similarity between the physiological function of promoting neuronal inhibition and the mental function of tranquillizing.

## Inadequacy of the ‘Covering Law’ Model

The processes studied by Newtonian mechanics were of the right kind to be captured and predicted by deductive procedures, but the emergence of mental from biophysical relations seems to involve a kind of process that cannot be captured and predicted in a deductive framework. What is the reason of this possible limitation? Is it because the emergence process follows an intrinsically probabilistic instead of a deterministic path? Is the link between biophysical and mental structures a mathematical function (one-to-one or many-to-one mapping of first-order relations) that can be deductively computed or merely a relation (that also includes one-to-many mappings) that would require more sophisticated computing strategies?

The classical deductive-reductionist explanatory strategy is based on the assumption that the reduced theory (putatively expressing the dynamics of the phenomenon to be explained) can be completely derived from the reducing theory, eventually complemented by bridge principles and the initial and boundary conditions. Such a bias is historically grounded on Newtonian mechanics, where three single laws were believed to completely explain the movement of all physical bodies. Such an explanatory power was based on a methodological simplification of the system, by ignoring friction, heat dissipation, many-body interactions, non-linear feed-back effects and other complex features of the real physical world.

This kind of deductive explanation, also known as the “Covering Law” model, has been criticized in recent approaches to complex systems. It was not well accepted in Biology, an area where the knowledge of structural properties is usually considered to have more explanatory power than the knowledge of laws. The reason derives from evolutionary theory. There are two random factors that hinder purely deductive approaches to evolutionary processes: the first one relates to the generation of biological novelty by means of mutations and the second relates to the allopatric speciation mechanisms proposed by Ernest Mayr, which involves a random geographical isolation that bypasses Hardy-Weinberg populational statistics (for a detailed discussion see Hull, 1974).

A brief explanatory note on allopatric mechanisms and the Hardy-Weinberg law would be appropriate here. Allopatric speciation is a process whereby a small group belonging to a biological population is geographically (and, as a consequence, also genetically) isolated from the larger group. In this condition, fluctuations in the frequence of genes, caused by mutations in one or more individuals, can be amplified and eventually dominate the isolated sub-group. In a larger population, such an individually originated fluctuation could not become dominant because of the Hardy-Weinberg law, that establishes the form of statistical calculation of the frequency of genes in populations. According to this statistical law, small fluctuations compensate and therefore cancel each other, not influencing the average values that dominate in the system as a whole.

In the Philosophy of Mind, with greater reason, the deductive-reductionist approach has limitations. The brain-body-environment system involves complex interactions between its components, generating results that apparently cannot be predicted from law-like sentences that compose current scientific theories.

In this situation, one possibility is to abandon the deductive-reductionist approach and make use of an alternative form of scientific explanation, such as searching for *similarities* between biophysical and mental processes. Possibly this alternative approach to scientific explanation can afford a new approach to the central issues debated in the Philosophy of Mind, allowing philosophers to find less ambitious - but more realistic - solutions to the problem of understanding the relation of brain and mind.

One development in this direction is the new paradigm of *embedded and embodied cognition.* “Embedded” means cognitive processes should be considered in a context, which - for human beings - includes historical-cultural constraints. “Embodied” means the cognitive agent operates in a physical/biological body that participates in the cognitive processes. In this paradigm, old assumptions made by AI about cognition as a purely logical process are abandoned. This conceptual move allows some old problems faced by traditional computational approaches - as the so-called “frame problem” - to be solved by means of an immersion of the cognitive system in an informationally rich environment, dispensing it from the necessity of holding all relevant information in its internal memory. This conceptual move leads to the revaluation of commonsense knowledge and inductive/abductive reasoning as alternatives to overcome the difficulties found in the deductive approach.

## Concluding Remarks

Structuralism is an alternative, besides reductionism and eliminative materialism, that provides inductive explanation of psychological functions from biophysical theories and data, integrating the results of molecular neurobiology with cognitive neuroscience.

Philosophically, I propose that mental activity emerges from the organization and activity of elements and mechanisms of nature, which determine the range of possibilities of our consciousness. In this view, brain organization and activity are important (and probably necessary) to make possible the manifestation of mental life. This *structuralist-naturalistic* approach overcomes the dualism of the physical and the mental, while at the same time avoids *panpsychism* - the philosophical theory that attributes mental life to all aspects of nature. Only the elements and mechanisms of nature that participate in brain activity supporting consciousness are allowed to lend their mark into the process.

In this perspective, mentality, as well as the divine dimension of reality (the Infinite, as proposed in the philosophy of Spínoza), are embedded in physical nature and become manifest only by means of physical elements and mechanisms. In such an immanentist and monist view of reality, the Infinite is manifested only through the Finite aspects of nature that we experience. The Cognitive Neurosciences, as a new research area, can be considered as a limited step in the direction of such a naturalistic worldview. However, it is a very important one, since it obeys standard procedures of scientific investigation and has been accepted in the context of western science and culture.

The progress of scientific knowledge usually does not give a definitive answer to philosophical problems, but can impel scientifically-informed philosophers towards a better formulation (or reformulation) of fundamental issues. The strong correlation between brain activity and mentality found in the Cognitive Neurosciences can change the center of philosophical inquiry about the relation of brain and mind: instead of asking *if* there is such a relation, we are impelled to ask *how* this relation occurs. The answer to the newer question may take a long time to appear, since it requires from us a fuller understanding of how the brain works. The Cognitive Neurosciences have provided us with good empirical demonstrations about the relation between brain activity and mentality, but we are still far away from explaining how the brain generates (or is generated by) the mind.

### Take home message

The progress of science can help to solve philosophical problems, but not automatically. The decision to take scientific results seriously in ontological matters is itself a philosophical decision. There are several possible philosophical interpretations of scientific results related to the philosophical mind-brain problem. In my view, the deepening of scientific research into the fundamental dimensions of nature, from the molecular to the quantum level of organization, can be a source of important insights about who we are, why we exist and what we are doing in this world, just to mention some basic philosophical issues. On the other hand, for those who place the human mind in a domain of reality separated from nature, the knowledge of brain mechanisms cannot possibly throw new lights upon the human mind.

## Questions that the paper raises

- Do the Cognitive Neurosciences provide a non-reductionistic approach to the human mind? Is Molecular Neurobiology intrinsically reductionist? Or does the judgment depends on our own worldview?
- Can the standards of Western scientific methodology allow for an understanding of the human mind? Is it possible to understand human consciousness as a collection of objective, measurable processes in the brain?
- How much would Psychiatry benefit from the Cognitive Neurosciences? Does it provide a broader view of the brain/mind than molecular neurobiology? Could it help to formulate new therapeutic directions?
- What is the place of the human mind and spirituality in the world? Are the mind and the divine dimension of reality inside and/or outside Nature? Does investigation of the smallest parts of nature bring us closer to or take us more distant from, the spiritual dimension of reality?
- If Naturalistic Structuralism is true, what are the brain mechanisms that manifest mentality and spirituality? Could they be explained by quantum theory?

## About the Author

Alfredo Pereira Junior has done his Masters in Philosophy (Federal University of Minas Gerais, 1986) and Doctorate (PhD) in Logic and Philosophy of Science (State University of Campinas, 1994). He was a Post-Doctoral Fellow in Sciences of the Brain and Cognition at the Massachusetts Institute of Technology (1996-98). Currently he is Adjunct Professor at the State University São Paulo Júlio de Mesquita Filho. He has experience in the area of Philosophy, with emphasis in Philosophy of Science, related to the following subjects: consciousness, brain science, cognitive neuroscience, molecular neurobiology and philosophy of science.


